# Differential expression of cardiometabolic and inflammation markers and signaling pathways between overweight/obese Qatari adults with high and low plasma salivary α-amylase activity

**DOI:** 10.3389/fendo.2024.1421358

**Published:** 2024-10-01

**Authors:** Olfa Khalifa, Neyla S. Al-Akl, Abdelilah Arredouani

**Affiliations:** ^1^ Diabetes Research Center, Qatar Biomedical Research Institute, Hamad Bin Khalifa University, Qatar Foundation, Doha, Qatar; ^2^ College of Health and Life Sciences, Hamad Bin Khalifa University, Qatar Foundation, Doha, Qatar

**Keywords:** cardiometabolic disease, low-grade inflammation, proteomics, salivary α-60 amylase activity, Olink

## Abstract

**Background:**

The relationship between salivary α-amylase activity (sAAa) and susceptibility to cardiovascular disorders lacks a definitive consensus in available studies. To fill this knowledge gap, the present study endeavors to investigate this association among overweight/obese otherwise healthy Qatari adults. The study specifically categorizes participants based on their sAAa into high and low subgroups, aiming to provide a more comprehensive understanding of the potential link between sAAa levels and cardiovascular and inflammation markers in this population.

**Methods:**

Plasma samples of 264 Qatari overweight/obese (Ow/Ob) participants were used to quantify the sAAa and to profile the proteins germane to cardiovascular, cardiometabolic, metabolism, and organ damage in low sAAa (LsAAa) and high sAAa (HsAAa) subjects using the Olink technology. Comprehensive statistical tools as well as chemometric and enrichments analyses were used to identify differentially expressed proteins (DEPs) and their associated signaling pathways and cellular functions.

**Results:**

A total of ten DEPs were detected, among them five were upregulated (QPCT, LCN2, PON2, DPP7, CRKL) while five were down regulated in the LsAAa subgroup compared to the HsAAa subgroup (ARG1, CTSH, SERPINB6, OSMR, ALDH3A). Functional enrichment analysis highlighted several relevant signaling pathways and cellular functions enriched in the DEPs, including myocardial dysfunction, disorder of blood pressure, myocardial infraction, apoptosis of cardiomyocytes, hypertension, chronic inflammatory disorder, immunes-mediated inflammatory disease, inflammatory response, activation of leukocytes and activation of phagocytes.

**Conclusion:**

Our study unveils substantial alterations within numerous canonical pathways and cellular or molecular functions that bear relevance to cardiometabolic disorders among Ow/Ob Qatari adults exhibiting LsAAa and HsAAa in the plasma. A more comprehensive exploration of these proteins and their associated pathways and functions offers the prospect of elucidating the mechanistic underpinnings inherent in the documented relationship between sAAa and metabolic disorders.

## Introduction

The predominant protein identified within human saliva is salivary α-amylase (sAA), a calcium-dependent metalloenzyme integral to the initiation of the hydrolysis process for starchy substances within the oral cavity (1). The *AMY1* gene regulates the expression of sAA (2). A similar α-amylase, termed pancreatic α-amylase and regulated by the *AMY2* gene (3), is synthesized by the pancreas to conclude the hydrolysis of α-polysaccharides within the small intestine (4). The *AMY1* gene in the human genome exhibits notable Copy-Number Variation (CNV), with a range spanning from 2 to 20 copies (5–7). CNV is characterized by a deviation in the number of copies of a specific gene from that observed in a reference genome, resulting from the deletion or duplication of DNA regions (8).

While a positive correlation has been established between AMY1 copy number (CN) and sAA protein levels and enzymatic activity (2, 9–11) certain studies propose that the contribution of AMY1 CN to the expression and sAAa is limited (9, 12, 13).

Several investigations have revealed that AMY1 CNV and sAAa are inversely associated with obesity and insulin resistance (IR) (6, 14–18). However, other studies failed to observe such associations (8). In the adult Qatari population, recently reported an association between elevated sAAa and diminished odds of obesity (6) as well as a reduced likelihood of diabetes in women (7).

The prevalence of metabolic and cardiovascular disease (CVD) has surged within the Middle Eastern region, particularly in Qatar, over the preceding two decades (19, 20). According to the last annual report (2022-2023) from the Qatar Biobank (QBB), which enrolls participants from the general population, 35.3% (20% men) and 41% (18% men) of the adult Qataris are overweight and obese, respectively (https://researchportal.qatarbiobank.org.qa/login). Furthermore, the same report shows that type 2 Diabetes (T2D) afflicts approximately 15.5% of the adult individuals, almost double the global prevalence (19). Consequently, a pressing imperative arises to gain comprehensive insights into the fundamental pathophysiological mechanisms of these conditions and identify predictive biomarkers that could help implement preventive strategies. Given that both obesity and T2D are strong risk factors for CVD, research is particularly salient in the context of elucidating markers of increased risk of CVDs.

In our recent investigations, we explored the correlation of sAAa with obesity and diabetes within the general population of Qatar without focusing on any specific group of individuals. However, in consideration of the established association between sAAa and obesity and recognizing the robust association of obesity with several comorbidities especially CVD, it is worthwhile to a focus on overweight or obese (Ow/Ob) individuals who are at high-risk of CVD events. Such a focus may yield valuable insights into the intricate relationship between sAAa levels and markers of cardiometabolic risk.

The aim of this study was to investigate the relationship between sAAa levels and markers of cardiovascular and inflammation in Ow/Ob Qatari individuals. Specifically, participants were categorized into LsAAa and HsAAa subgroups to better understand how variations in sAAa levels correlate with these markers. This cross-sectional study utilizes the highly sensitive Olink Proximity Extension Assay (PEA) technology to quantify proteins from individuals who are overweight or obese but otherwise in good health.

## Methods

### Study design, participants, and plasma collection

Plasma samples and clinical data of 264 Ow/Ob, normoglycemic participants were used in this cross-sectional study. The 264 subjects were selected from a larger cohort of 1500 individuals, in which we had previously quantified the sAAa activity (21). Clinical, demographic, and anthropometric data of the 1500 subjects were obtained from the Qatar Biobank (22). A well-phenotyped cohort of adults from the general Qatari population was utilized. All participants were normoglycemic, with the following eligibility criteria: aged ≥18 years, fasting for ≥6 hours at the time of specimen collection, and having a BMI ≥ 25 kg/m². Pregnant women, individuals with a BMI <25 kg/m², and those with diabetes (HbA1c ≥ 6.5%) were exclusion from the study. Detailed information regarding their selection process and characteristics is available in the QBB.

### Consent and ethical approvals

All participants within the QBB cohort have formally provided their consent through the execution of an informed written agreement, granting authorization for the utilization of their data and biospecimens in the pursuit of research endeavors. Approval for the current study has been obtained from the institutional review boards of both QBB (IRB number: Ex-2017-RES-ACC-0054-0018) and QBRI (IRB number: 2017–001).

### Quantification of the sAAa in plasma

The quantification of sAAa was executed on plasma samples utilizing an enzymatic colorimetric assay performed with an autoanalyzer (ARCHITECT c4000; kits #6K22-30 and #7D58-21; ABBOTT Laboratories, Bluff, Illinois, USA). The methodology encompassed two distinct reactions designed to assess the enzymatic activities of both total α-amylase (tAA) and pancreatic α-amylase (pAA). The determination of sAAa was derived by deducting the pAA activity from the tAA activity. This assay was externally contracted to Micro Health Laboratories, a private medical laboratory located in Doha (https://www.microhealthcare.com).

### Proteomics and Olink assays

**Figure 1:**
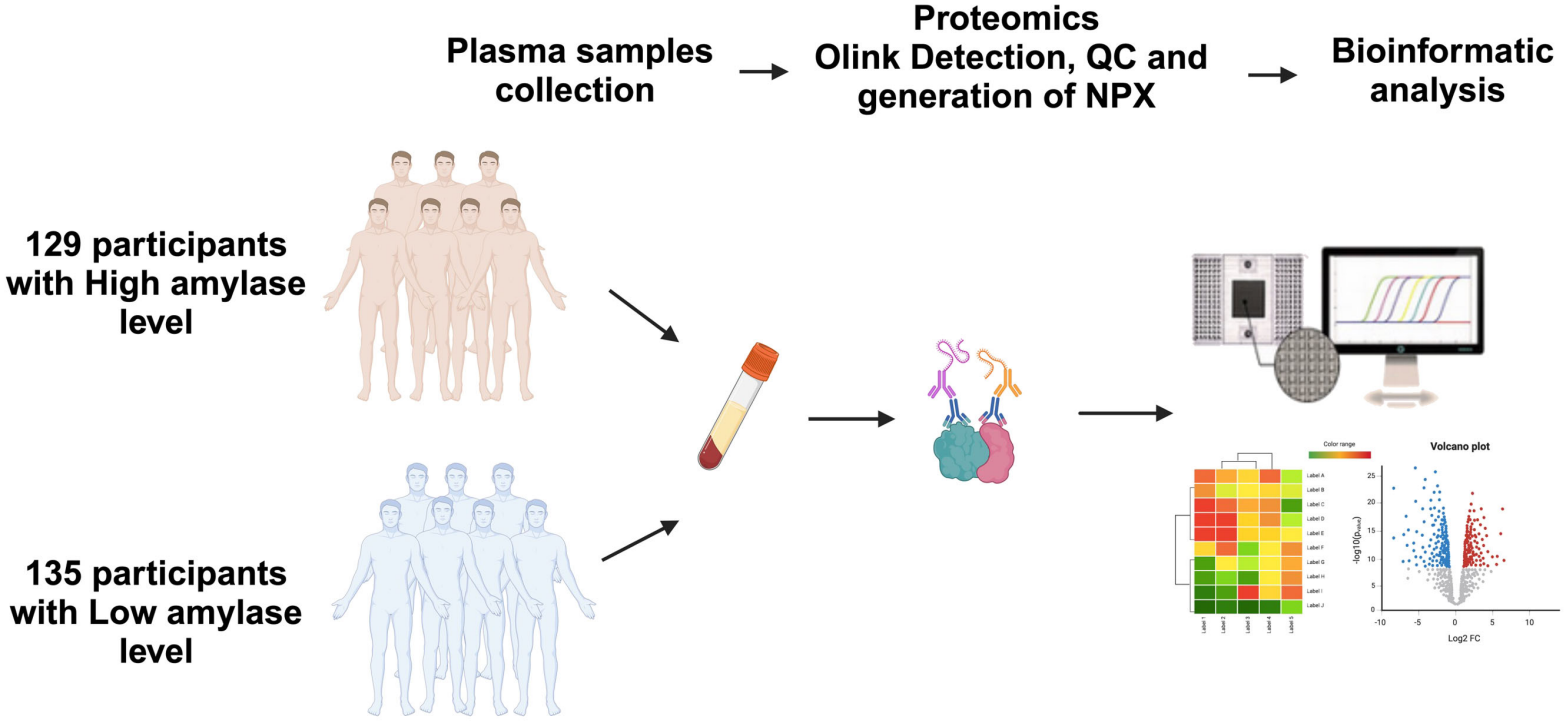
Study design and schematic illustration of plasma Olink proteomics. Plasma samples from participants with high and low salivary α-amylase activity (sAAa) n=129 vs n=135 Qatari population were collected. The matrix for different panels for Olink technology was prepared. Many bioinformatics tools were employed to analyze and display the differentially expressed proteins (DEPs).

Plasma proteins were profiled using the Olink PEA, 92-plex immunoassay (Uppsala, Sweden) (17) following the standard protocol at the Qatar Biomedical Research Institute’s (QBRI) Olink certified proteomics core facility. Quality control and data normalization were carried out using the Normalized Protein eXpression (NPX) software and every run was checked and validated by the Olink support team in Uppsala, Sweden. Five different panels focusing on cardiovascular biomarkers (Olink Target 96 Cardiovascular II, #95500, Uppsala, Sweden, Olink Target 96 Cardiovascular III, #95611, Uppsala, Sweden), cardiometabolic panel (Olink Target 96 cardiometabolic, #95360, Uppsala, Sweden), metabolism panel (Olink Target 96 Metabolism, #95340, Uppsala, Sweden) and organ damage (Olink Target 96 Organ Damage, #9533, Uppsala, Sweden) were used in our study. Protein expression values were calculated as log2 (NPX), and Olink data that did not pass quality control were excluded from the analyses. Starting with the pre-processed NPX data Olink NPX Manager (version 2.1.0.224), protein differential expression analysis was performed using different statistical methods. **Figure 1** illustrates the flow of the proteomics profiling and analysis.

### Anthropometric and clinical measures

The central laboratory in Hamad Medical Corporation in Doha carried out all clinical measurements. Body composition was determined by Bioimpedance analysis (Tanita). The visceral adiposity index (VAI), body fat percentage (BFP), fat mass index (FMI), and body adiposity index (BAI) for both men and women were computed using the formula provided below:


$$\begin{array}{l} VAI\;\left(women\right)=\;[\left(WC\;\left(in\;cm\right)/36.58\right)\;+\;(1.89\;\\ \;\times \;BMI\;\left(in\;Kg/m2\right))]\;\\ \;\times \;[\left(TG\;\left(in\;mmol/L\right)/0.81\right)\;\\ \;\times \;\left(1.52/\text{HDL-C }\left(in\;mmol/L\right)\right)]\left\{Dong,\;2021\;\#159\right\} \end{array}$$

Body Fat %=Absolute Fat mass/weight (Kg)∗100

Fat Mass Index=Absolute Fat mass/Height (m)

$$\begin{array}{c} Body\;Adiposity\;Index\\ \;=hip\;circumference\;\left(cm\right)/height\;\left(m\right)1.5–18\;\\ \;\left\{Freedman,\;2012\;\#160\right\}\;(Bergman\;et\;al.,\;2011) \end{array}$$

### Determinant and network analyses

To assess potential distinctions in proteomic profiles between individuals with LsAAaand high (HsAAa) sAAa levels, orthogonal projections to latent structures discriminant analysis (OPLS-DA) was employed on the extreme 25% values of the two groups. Proteins exhibiting a Variable Importance in Projection (VIP) score exceeding 1, a commonly employed threshold for feature relevance, were deemed significant contributors to the observed separation. Subsequently, these identified proteins underwent analysis using Ingenuity Pathway Analysis (IPA) from QIAGEN (Redwood City, CA, USA) to discern specific enriched networks and pathways. Assessment of protein-protein interactions was conducted using STRING (https://string-db.org/; accessed on 5 October 2023).

### Statistical analyses

All statistical analyses were performed using GraphPad Prism 9.0 software (GraphPad Prism v9, La Jolla, CA, USA). Independent samples t-test was used for the comparison of continuous variables between groups. OPLSDA was performed on Metaboanalyst (https://www.metaboanalyst.ca/). Correlations were examined with the Pearson coefficient between two variables using GraphPad Prism 9.0 software. Simple linear regression was used to investigate the association between continuous variables. A p-value <0.05 was considered statistically significant.

## Results

### Characteristics of study participants

The sAAa levels within the cohort of the 264 participants exhibited a range spanning from 13.5 to 94 U/L. The mean sAAa concentrations were 15.6 (±4) U/L and 69.2 (±12) U/L in the LsAAa and HsAAa groups, respectively. To enhance the likelihood of discerning a statistically significant distinction in proteomic profiles between the studied groups, our investigation was confined to the upper quartile (top 25%) of the HsAAa group and the lower quartile (lower 25%) of the LsAAa group. Basic characteristics of the extreme 25% of each group are presented in **Table 1**. Most participants in the LsAAa and HsAAa subgroups were females (87.8% and 62.5%, respectively). The mean age was 36.4  ± 12 and 38.5  ± 11 years in the LsAAa and the HsAAa subgroup, respectively. As shown in **Table 1**, only total proteins, total bilirubin, creatinine, and insulin levels were significantly different between the LsAAa and HsAAa subgroups.

**Table 1:** Baseline clinical characteristics of the participants.

Clinical Characteristics	Low sAAa (n= 33)	High sAAa (n= 32)	*p*-value
**sAAa** (U/L)	15.6 (±4)	69.2 (±12)	**<0.0001**
**Age** (years)	36.4 (±12)	38.5 (±11)	0.4802
**Sex, male/female** (% women)	29/33 (88%)	29/32 (62%)	**0.0172**
**Height (cm)**	158 (±6.8)	162(±10)	**0.0400**
**Waist (cm)**	88.2 (±9.4)	89(±11)	0.6574
**Body weight (Kg)**	79.4 (±13)	81 (±14)	0.6087
**BMI (Kg/m2)**	31.8 (±4.5)	30 (±3.7)	0.2658
**WHR**	0.8 (±0.06)	0.8 (±0.08)	0.6567
**Total proteins (g/L)**	73.7 (±3.5)	76 (±3.9)	**0.0190**
**HBA1C %**	5.1 (±0.25)	5 (±0.3)	0.6582
**Total bilirubin**	7.8 (±3.2)	8.4 (±4)	**<0.0001**
**Triglycerides (mmol/L)**	1 (±0.4)	1 (±0.4)	0.3690
**Total Cholesterol (mmol/L)**	4.8 (±0.76)	4.8 (±0.8)	0.7715
**LDL Cholesterol (mmol/L)**	3 (±0.74)	2.8 (±0.7)	0.3824
**HDL Cholesterol (mmol/L)**	1.4 (±0.33)	1.5 (±0.3)	0.0981
**Glucose (mmol/L)**	5 (±0.5)	4.9 (0.4±)	0.8704
**Creatinine (mmol/L)**	58.2 (±8.6)	67 (±13.7)	**0.0036**
**Insulin (mmol/L)**	10 (±4)	12.3 (±5.2)	**0.0436**
**CRP (mg/L)**	7 (±8.9)	69.2 (±12)	0.2617
**ALT (mmol/L)**	22 (±27)	22 (±13)	0.9758
**AST (mmol/L)**	20 (±20)	19 (±6)	0.7818

sAAa, α-Salivary Amylase activity; BMI, Body Mass Index; WHR, Waist to Hip Ratio; HDL, High density lipoprotein; LDL, Low density lipoprotein; CRP, c-reactive protein; ALT, Alanine Aminotransferase; AST, Aspartate Aminotransferase; HbA1c, Glycated Hemoglobin. All values are reported as Mean ± SD. p-value highlighted in bold in Table.1 indicates a significant difference.

### Identification of DEPs

**Figure 2:**
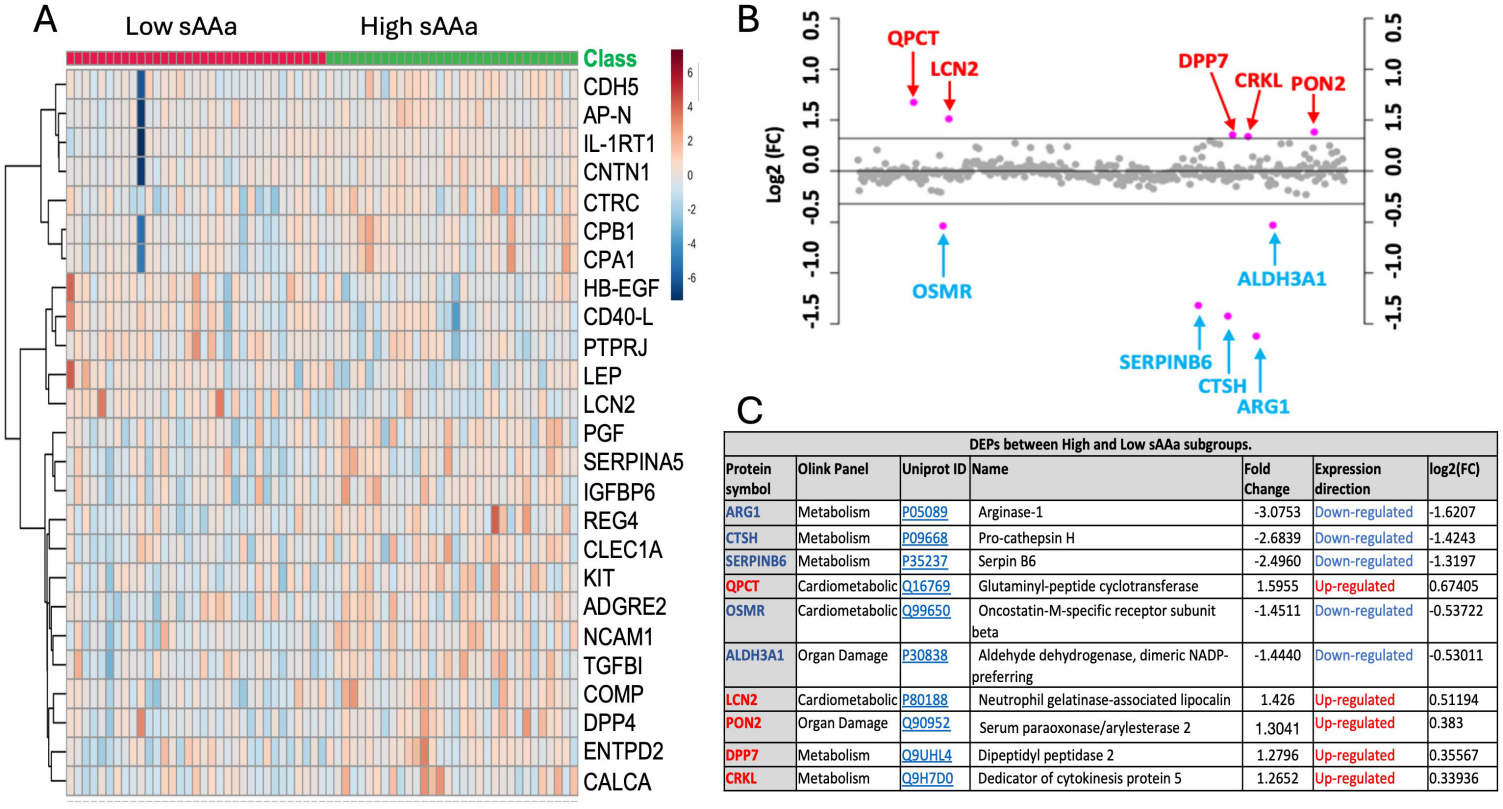
Identification of differentially expressed proteins (DEPs) in ow/ob nondiabetics Qatari population. **(A)** Heatmap of differentially expressed inflammation-related proteins. **(B)** Fold change between the low sAAa subgroup compared to the high sAAa subgroup (FC threshold= 1.25) based on the normalized protein expression (NPX). **(C)** 10 differentially expressed proteins (DEPs) were detected between the two subgroups. The up-regulated proteins were highlighted in Red, while the down- regulated proteins were in blue. The p-value was calculated using Student's t-test. Significant different proteins are shown in the table(p<0.05).

Different Olink panels were employed to detect differences in the expression of proteins among LsAAa and HsAAa individuals. Proteins are listed and enumerated in **Supplementary Table S1**. The heatmap in **Figure 2A** illustrates the hierarchical clustering of the top 25 proteins among the two groups. Under a fold change threshold of 1.25 and a significance level of 0.05, a total of 10 differentially expressed proteins (DEPs) were discerned. Specifically, five proteins—Glutaminyl-peptide cyclotransferase (QPCT), Neutrophil gelatinase-associated lipocalin (LCN2), Serum paraoxonase/arylesterase 2 (PON2), Dipeptidyl peptidase II (a.k.a. DPP7), and Dedicator of cytokinesis protein 5 (CRKL)—were found to be upregulated, while five proteins—Arginase-1 (ARG1), Pro-cathepsin H (CTSH), Serpin B6 (SERPINB6), Oncostatin-M-specific receptor subunit beta (OSMR), and Aldehyde dehydrogenase, dimeric NADP-preferring (ALDH3A1)—were observed to be downregulated in LsAAa samples LsAAa in comparison HsAAa samples (**Figures 2B, C**).

### Chemometric analysis

**Figure 3:**
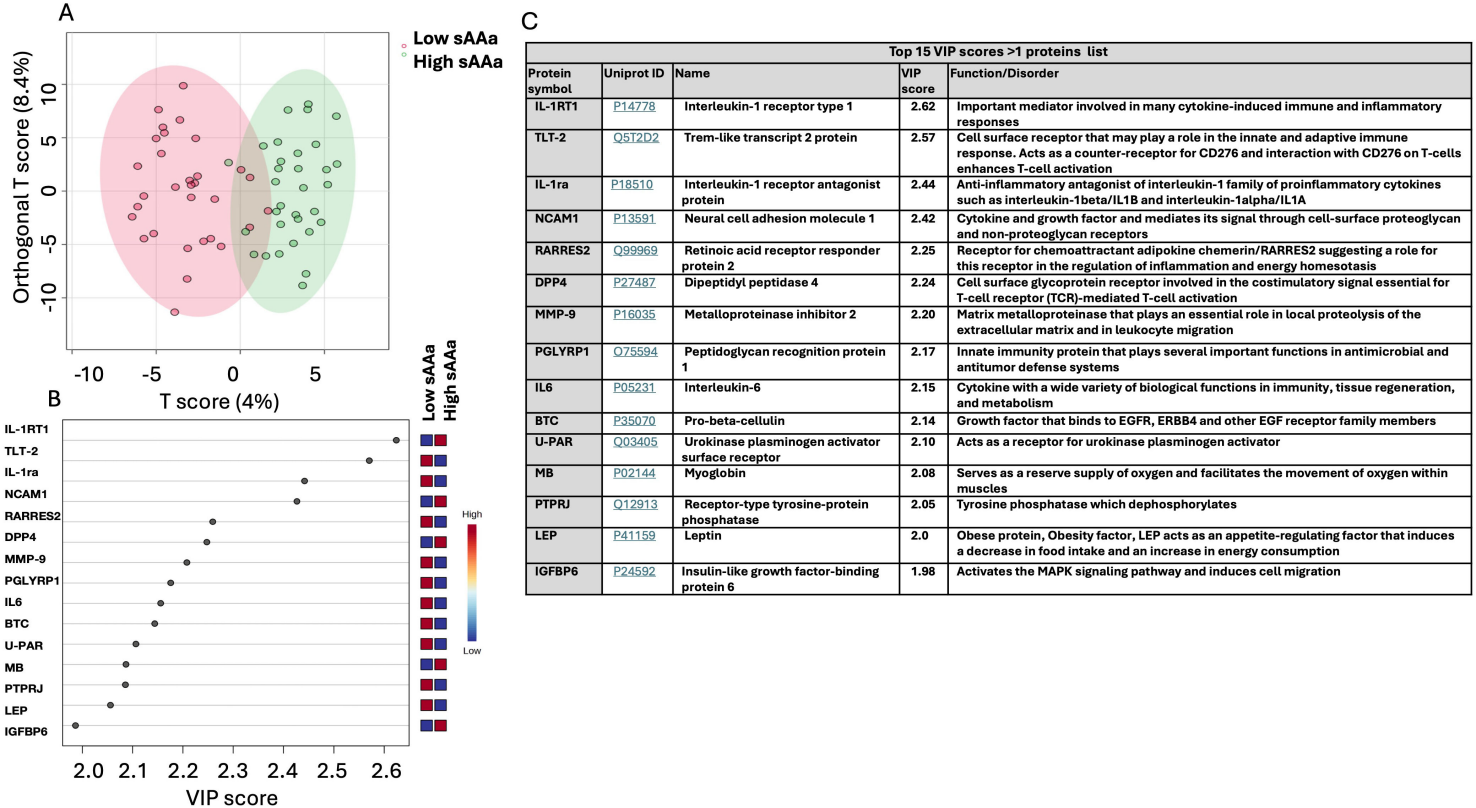
Chemometric analysis of proteins data set from the low sAAa samples compared to high sAAa samples. **(A)** The Orthogonal PLS-DA score plot between the low sAAa subgroup compared to the high sAAa subgroup. The colored oval shapes in A indicate the 95% confidence regions. **(B)** The important features (VIP scores) identified using Orthogonal PLS-DA analysis between the low sAAa subgroup compared to the high sAAa subgroup. The colored boxes on the right indicate the relative levels of the corresponding element in high sAAa and low sAAa samples. **(C)** Top 15 VIP scores >1 proteins list, Uniprot Id and protein symbol, their function and associated disorder is also detailed.

In conjunction with the analysis of DEPs, an Orthogonal Partial Least Squares Discriminant Analysis (OPLS-DA) was executed to delineate pivotal proteins facilitating the discrimination between the two groups, as illustrated in **Figure 3**. The score plot presented in **Figure 3A** demonstrates a notable demarcation between the participants belonging to the respective groups. To pinpoint the proteins predominantly contributing to the observed separation in **Figure 3A**, the Variable Importance in Projection (VIP) score was employed. Proteins with a VIP score exceeding 1 are conventionally regarded as significant for the observed separation. A total of 130 proteins meeting this criterion were identified, with the top 15 depicted in **Figure 3B**. **Figure 3C** presents the UniProt ID and functions corresponding to these 15 proteins. Among the 15 proteins, the Interleukin-1 receptor type 1 (IL-1RT1) protein exhibited the highest score with a VIP score of 2.62, while Insulin-like growth factor-binding protein 6 (IGFBP6) has the lowest impact on the demarcation between the two groups, with a VIP score of 1.98.

### Enrichment analysis

**Figure 4:**
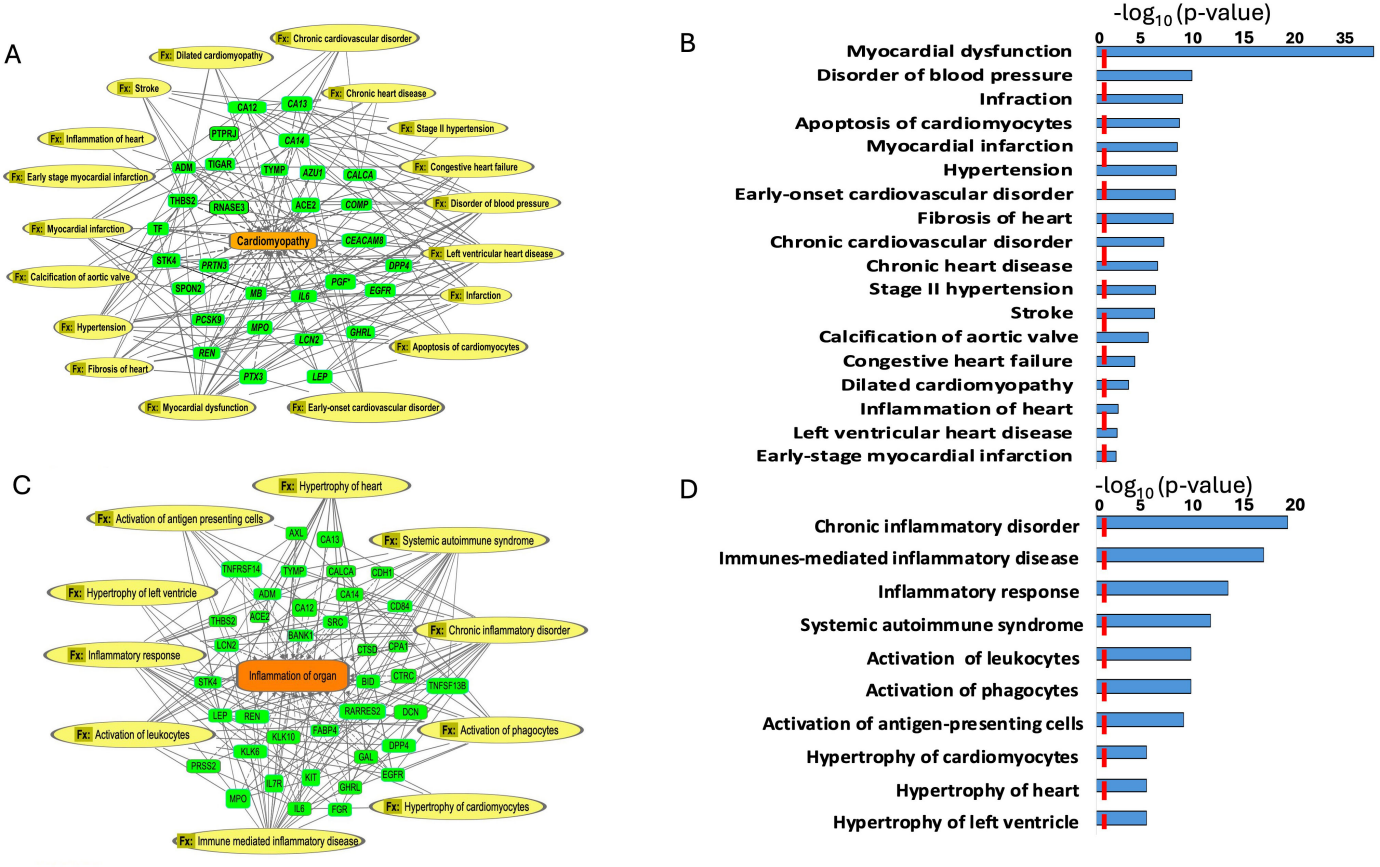
Enriched diseases and functions (Fx) network. **(A, B)** Enriched diseases and functions (Fx) using the important features (VIP scores) identified using Orthogonal PLS-DA analysis between the low sAAa subgroup compared to the high sAAa subgroup within cardiomyopathy. **(C, D)** Enriched diseases and functions (Fx) using the important features (VIP scores) identified using Orthogonal PLS-DA analysis between the low sAAa subgroup compared to the high sAAa subgroup within inflammation of the organ. The diseases and functions involve at least one protein. The diseases and functions involve at least one protein.

**Figure 5:**
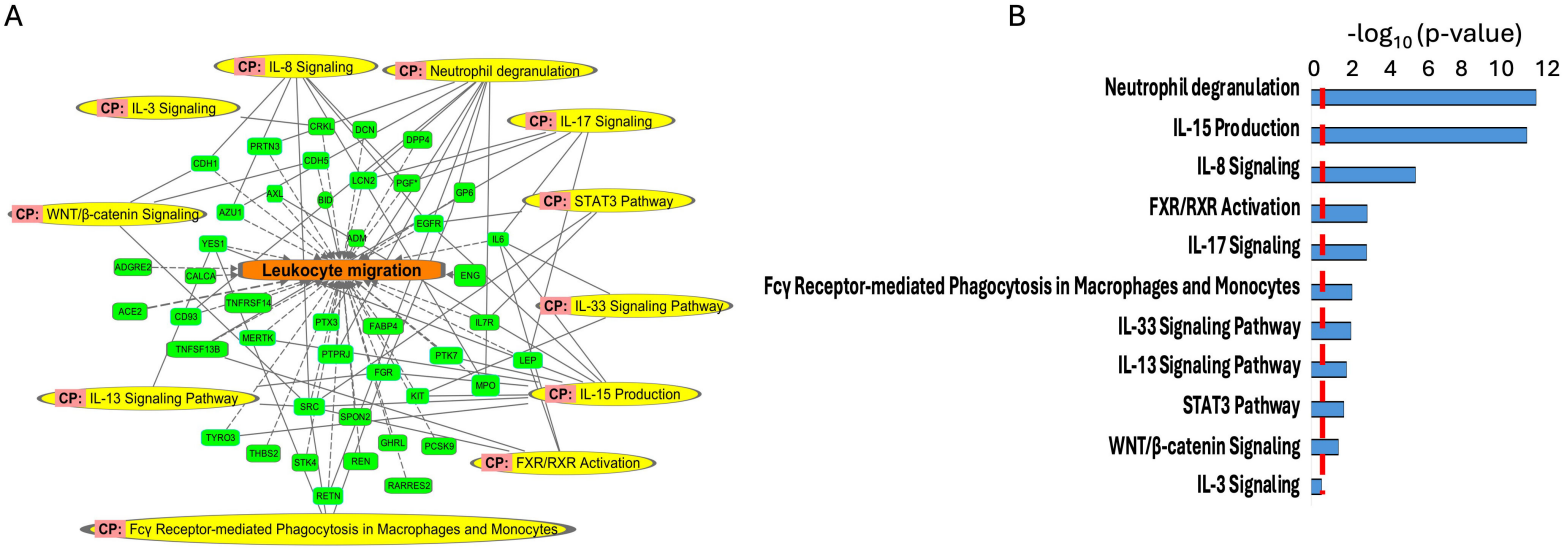
Enriched canonical pathways (CP) **(A, B)** using the important features (VIP scores) identified using Orthogonal PLS-DA analysis between the low sAAa subgroup compared to the high sAAa subgroup. The canonical pathways involve at least one protein.

To scrutinize the signaling pathways potentially implicating the DEPs between the LsAAa and HsAAa subgroups, proteins with a VIP>1, as discerned from the oPLS-DA analysis, were submitted to the Ingenuity Pathway Analysis application. This investigation identified 130 unique proteins targets (**Supplementary Table S1**) subsequently used them to determine the enriched canonical pathways, diseases, and cellular and molecular functions (**Figures 4**, **5**).

Our analysis identified several diseases and cellular activities linked to the DEPs potentially pertinent to cardiovascular disorders, inflammation and metabolic disease. Within Myocardial dysfunction, Disorder of blood pressure, Infraction, Apoptosis of cardiomyocytes, Myocardial infarction, Hypertension, Early-onset cardiovascular disorder, Fibrosis of heart, Chronic cardiovascular disorder, Chronic heart disease, Stage II hypertension, Stroke, Calcification of aortic valve, Congestive heart failure, Dilated cardiomyopathy, Inflammation of heart, Left ventricular heart disease,Early-stage myocardial infarction (**Figures 4A, B**) and inflammation including Hypertrophy of left ventricle, Hypertrophy of heart, Hypertrophy of cardiomyocytes, Activation of antigen-presenting cells, Activation of phagocytes, Activation of leukocytes, Systemic autoimmune syndrome, Inflammatory response, Immunes-mediated inflammatory disease, Chronic inflammatory disorder (**Figures 4C, D**). Meanwhile, we pinpointed numerous canonical pathways (CP) that were enriched in the DEPs proteins and that were relevant to metabolic and cardiovascular disorders, including Neutrophil degranulation, IL-15 Production, IL-8 Signaling, FXR/RXR Activation, IL-17 Signaling, Fcγ Receptor-mediated Phagocytosis in Macrophages and Monocytes,IL-33 Signaling Pathway,IL-13 Signaling Pathway,STAT3 Pathway,WNT/β-catenin Signaling,IL-3 Signaling (**Figures 5A, B**).

### Protein-protein interaction analysis

**Figure 6:**
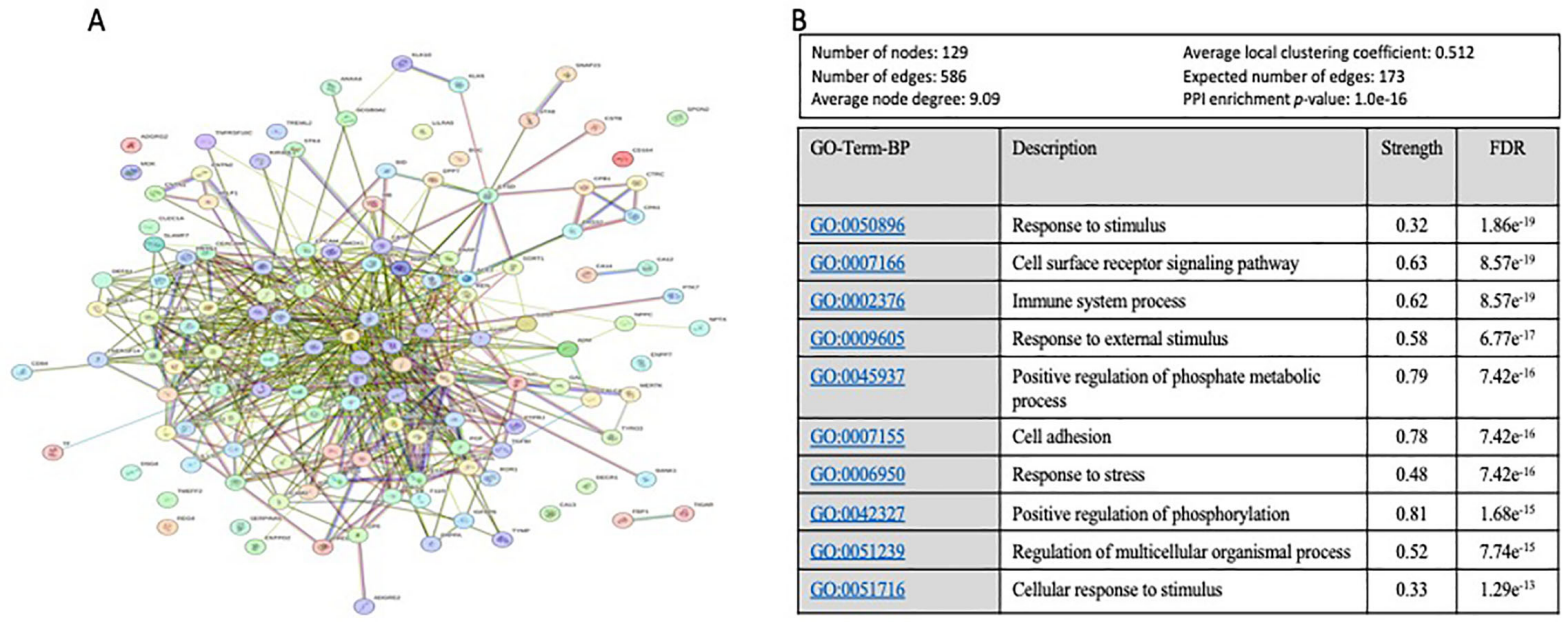
The protein-protein interaction (PPI) network and Top 10 of Gene Ontology (GO) Biological Process based on FDR. **(A)** The protein-protein interaction (PPI) network generated for the 130 proteins with a VIP score >1 proteins targets the identified DEPs between high and low aAAs among Qatari Ow/Ob but nondiabetic population. Network nodes represent proteins, while the edges depict protein-protein associations. The key network statistics are also presented. **(B)** The top 10 functional enrichment annotations from Gene Ontology (GO) Biological Process are listed. STRING Data Base 11.5 was used for data analysis.

**Figure 7:**
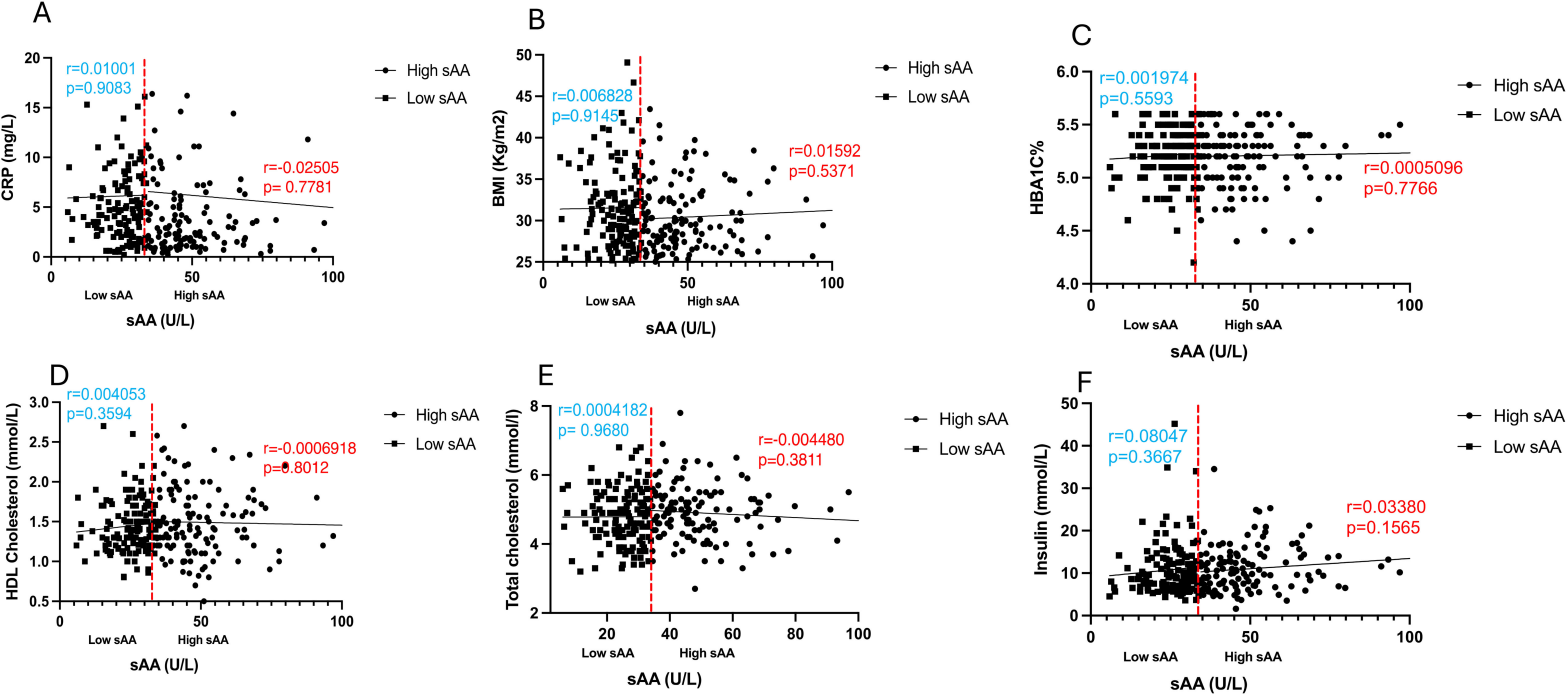
Scatter plots and best-fit lines depicting correlations between sAAa and different markers. CRP **(A)**, BMI **(B)**, HBA1C% **(C)**, HDL Cholesterol **(D)**, Total cholesterol **(E)**, Insulin **(F)**, in HsAA and LsAA subgroups. Red line represents the threshold between low and high sAA, dots represent participants with high sAA and squares represent participants with low sAA.

To further explore and annotate the potential molecular pathways affected by sAAa, DEPs was used to perform the protein-protein interaction (PPI) analysis using the STRING tool (23) (https://string-db.org/ accessed on 20 October 2023). As depicted in **Figure 6**, the PPT network revealed numerous noteworthy enrichments and associations among multiple proteins, indicating their involvement in mediating various pathways with FDR<0.05. There were 463 significant Gene Ontology (GO) terms for the biological processes. The top ten are shown in **Figure 6B** including: response to stimulus, cell surface receptor signaling pathway, immune system process, response to external stimulus, positive regulation of phosphate metabolic process, cell adhesion, response to stress, positive regulation of phosphorylation, regulation of multicellular organismal process, cellular response to stimulus. However, forty-four significant GO-terms were identified for molecular functions including: signaling receptor binding, glycosaminoglycan binding and signaling receptor activator activity. Finally, there were fifty-five significant GO-terms for cellular components such as extracellular region, cell surface, cell periphery and secretory granule. PPI analysis showed forty KEGG pathways significantly enriched among them cytokine-cytokine receptor interaction, cell adhesion molecules, MAPK signaling pathway, IL-17 signaling pathway. To analyze the relationships between sAAa and critical inflammation markers—CRP (A), BMI (B), HbA1c% (C), HDL Cholesterol (D), Total Cholesterol (E), and Insulin (F) in the high sAAa (HsAA) and low sAAa (LsAA) subgroups (**Figure 7**), our findings indicate that in participants with Ow/Ob, these markers do not show statistically significant differences between high and low activity subgroups. Additionally, we observed weak positive correlations between sAA and CRP, BMI in the Low sAA.

## Discussion

To the best of our knowledge, no previous studies have examined the differential expression of circulating proteins associated with cardiometabolic risk in Ow/Ob individuals with high and low sAAa. Leveraging Olink proteomics technology, chemometric, and enrichment analysis, the present cross-sectional study showed differential expression of multiple circulating plasma proteins between low and high sAAa individuals. Several of these DEPs are involved in various canonical pathways, as well as in cellular and molecular functions germane to cardiometabolic disease, including insulin secretion signaling pathway, NAFLD pathway, PPARAα/RXRα activation, NF-kB signaling, mTOR signaling, TR/RXR activation, and VEGF signaling, diabetes mellitus, insulin resistance, metabolic syndrome, concentration of cholesterol, steatohepatitis, glucose metabolism disorder, and hepatic steatosis.

The salivary alpha-amylase (sAA) is the predominant protein within saliva, and its enzymatic activity, along with the copy number of its encoding gene *AMY1*, exhibit an inverse association with various metabolic disorders, such as obesity and IR, across diverse populations of both children and adults (15). The precise mechanistic underpinnings of this association remain inadequately elucidated. Our team previously reported that normoglycemic and non-obese women harboring low copy number of the *AMY1* gene, hence low sAAa, shift their energy metabolism towards lipid utilization (24). This shift is probably attributable to a certain degree of insulin resistance, as indicated by elevated levels of 2-hydroxybutyrate, a discernible early marker of insulin resistance (15). Furthermore, Mandel et al. posited that individuals characterized by diminished sAAa exhibit aberrant glucose response after starch ingestion, presumably stemming from an impairment in cephalic phase insulin secretion (25). Further investigations are warranted to elucidate the mechanisms behind the observed inverse associations fully.

Numerous investigations examined the relationship between sAAa and metabolic disorders but predominantly focusing on individuals of the general population rather than specifically targeting Ow/Ob subjects who exhibit an elevated susceptibility to cardiometabolic disorders (26–29). The identification of differentially expressed circulating proteins between Ow/Ob subjects with low and high sAAa levels holds the potential to elucidate the underlying mechanistic pathways associating low sAAa with an augmented risk for obesity and, consequently, predisposition to a spectrum of cardiometabolic disorder, encompassing CVD, stroke, T2D, IR, and NAFLD.

The hematologic tests of the blood samples of the participants used in this study revealed a statistically significant elevation in total bilirubin levels among HsAAa individuals compared to their LsAAa counterparts. Previous investigations have demonstrated an inverse correlation between total bilirubin concentrations and the risk of various cardiometabolic disorders, notably CVD (30), hypertension (31) and stroke (32, 33). It is worth noting that HsAAa was associated with a diminished risk of obesity, a recognized high-risk factor for these cardiometabolic pathologies.

Our analysis identified significant cardiometabolic disease-associated DEPs between LsAAa and HsAAa samples. For instance, Arginase1, an enzyme that plays a crucial role in the urea cycle (34) is downregulated in LsAAa relative to HsAAa samples. By regulating various cellular functions and processes, including senescence, apoptosis, proliferation, inflammation, and autophagy, Arginase1 plays vital roles in the pathogenesis of cardiometabolic diseases, such as hypertension, atherosclerosis, stroke, ischemic reperfusion injury, and heart failure (35). Pro-cathepsin H (CTSH) is also downregulated in LsAAa samples. Its cleavage yields the enzyme cathepsin H, an aminopeptidase important for proteolysis and some specific reactions in the endosomal-lysosomal compartment. Overexpression of the CTSH has been detected in atherosclerotic lesions in co-localization with modified LDL (E-LDL) (36). A diminished soluble Oncostatin M receptor (sOSMR) expression is evident in LsAAa compared to HsAAs samples. Oncostatin M (OSM), a member of the interleukin-6 family, assumes a pivotal role as a mediator in remodeling cardiomyocytes within pathological contexts. Its implication extends to various human cardiac disorders, such as aortic stenosis, myocardial infarction, myocarditis, cardiac sarcoidosis, and diverse cardiomyopathies (37). Moreover, reduced sOSMR levels in individuals with coronary artery disease may significantly contribute to the pathophysiological mechanisms underlying the condition. Additionally, a correlation exists between diminished sOSMR levels and hypertension (38). Notably, murine studies have elucidated that the OSMR knockout exacerbates pressure overload-induced cardiac hypertrophy by influencing macrophages and the OSM/LIFR/STAT3 signaling pathway (39). The secreted glutaminyl-peptide cyclotransferase (QPCT) is amongst the proteins upregulated in LsAAa samples. QPCT catalyzes a post-translational chemical reaction in proteins or peptides that converts N-terminal glutamine or glutamate residues into N-terminal pyroglutamate (pE) by releasing ammonia or a water molecule, respectively (40). Recently, Karason et al. investigated heart failure in obesity using proteomics profiling in patients treated with or without weight-loss surgery (41) and identified eleven proteins associated with increased heart failure risk, including QPCT that had an odds ratio of 3.37 (p<0.001). LCN2 is also upregulated in LsAAa samples. LCN2 is an osteoblast-secreted protein that suppresses appetite and decreases fat mass while improving glucose metabolism (42). It has been shown that LCN2 is upregulated during obesity and diabetes as a protective mechanism to counteract obesity-induced glucose intolerance by decreasing food intake and promoting adaptive β-cell proliferation (42). A recent study has also suggested that circulating LCN2 may be protective against obesity and T2DM (43). Furthermore, LCN2 in the intestinal tract regulates the composition of the gut microbiota and shows anti-inflammatory activities (44). The upregulation of LCN2 in LsAAa samples could be a protective mechanism against the risk of obesity in these individuals. The serum paraoxonase/arylesterase 2 (PON2) is also upregulated in LsAAa samples. A constituent of the paraoxonase gene family, PON2 demonstrates ubiquitous expression and localization within the nuclear envelope, endoplasmic reticulum, and mitochondria. Noteworthy is its capacity, upon overexpression in cells, to mitigate endoplasmic reticulum (ER) stress, curtail mitochondrial superoxide formation, inhibit cardiolipin peroxidation, and suppress apoptosis (45). Conversely, reducing PON2 levels in cellular contexts results in heightened ER stress (45). Insight from murine models underscores the consequential impact of PON2 deficiency, culminating in augmented atherosclerosis attributable to diminished anti-oxidative and anti-inflammatory capabilities (46). Additionally, recent findings by Shih et al. underscore the association between PON2 deficiency and heightened susceptibility to diet-induced obesity (45). The exploration of PON2’s role in cardioprotection reveals its potential in mitigating heart failure, both in experimental models and human contexts, posited to stem from its adeptness in enhancing mitochondrial function and attenuating reactive oxygen species (ROS) generation (47). An upregulation in the expression of DPP7 was additionally noted in LsAAa samples. DPP7 belongs to the proline-specific dipeptidyl peptidases (DPPs) family, which significantly influence the modulation of peptide hormone signaling and participate in metabolic processes pertinent to diabetes, oncology, and hematology (48). DPP2 localizes to the vesicular compartment of the cytosol (49), but a recent study identified circulating DDP7 in samples from individuals with Prader-Willi syndrome and hepatic steatosis (50). Interestingly, a conditional knockout mouse model deficient in DDP7 exhibited hyperinsulinemia, impaired glucose tolerance, insulin resistance, enhanced liver steatosis, and visceral obesity (51). Therefore, the high levels of DDP7 in LsAAa individuals might represent a protective mechanism against metabolic disorders associated with LsAAa.

Chemometric analysis has uncovered the presence of significant proteins that assume a pivotal role in separating individuals with extreme levels of low and high sAAa. Subsequent enrichment and pathway analyses utilizing these proteins have demonstrated the enrichment of various canonical pathways linked to cardiometabolic diseases. Illustratively, the insulin secretion signaling pathway emerges as a central orchestrator of glucose and lipid metabolism, with alterations in this pathway bearing direct consequences for the initiation and progression of cardiometabolic diseases, including obesity, T2D, and dyslipidemia. Such perturbations may subsequently give rise to diverse complications such as cardiovascular disease (CVD), stroke, and NAFLD (52).

Another notable pathway in our analysis pertains to the activation of PPARAα/RXRα. PPARs and RXRs are important ligand-activated transcription factors that modulate gene expression. The PPAR/RXR transcriptional complex assumes a pivotal role in maintaining energy equilibrium, particularly governing triglyceride metabolism, fatty acid processing and storage, as well as glucose homeostasis. Dysregulation of these processes underlies the pathophysiology of obesity, T2D, and atherosclerosis (53).

The NF-κB signaling pathway also emerges as significantly enriched in our analysis. The transcription factor NF-κB, a master regulator of innate and adaptive immune responses, inflammation, cell proliferation, and apoptosis, is implicated in the development and progression of various cardiometabolic disorders, including obesity, T2D, and CVD (54). Several studies suggest that NF-κB activation may contribute to the chronic low-grade inflammation (55, 56) observed in obesity and insulin resistance, key factors in the development of T2D. Moreover, NF-κB activation has been linked to atherosclerosis (57, 58) a condition underlying many cardiovascular diseases.

The mTOR signaling pathway is also enriched in our comprehensive analysis, with mTOR activation implicated in a spectrum of pathological conditions, including obesity, diabetes, and CVD (59–61). Another pivotal pathway identified is the VEGF signaling pathway, critical in angiogenesis (62). Perturbations in VEGF signaling have been associated with various cardiometabolic disorders, including diabetes and its complications (63, 64), hypertension (65, 66), and CVD (67, 68). Our analysis further reveals enrichment in the TR/RXR activation pathway, where disruptions may impact metabolic health, contributing to cardiometabolic disorders, including obesity, insulin resistance, and metabolic syndrome (69–71). Additionally, the NAFLD pathway is also enriched, and recent clinical evidence underscores its direct association with an increased risk of cardio-metabolic disorders, including stroke, arterial hypertension, T2D, dyslipidemia, cardiac arrhythmias, and chronic kidney disease (72–76).

Besides the canonical pathways, our analysis has also unveiled enrichment in various diseases and functions, including diabetes mellitus, insulin resistance, metabolic syndrome, concentration of cholesterol, steatohepatitis, glucose metabolism disorder, and hepatic steatosis. All these conditions are intricately linked to cardiometabolic health.

Proteins identified as significant contributors to the delineation between High and low sAAa samples manifest intricate interconnections across diverse levels of interaction. This resultant network, comprising 129 nodes, is principally engaged in various physiological processes encompassing external and cellular responses to stimuli, positive regulation of phosphate metabolic processes, immune system modulation, cell surface receptor signaling pathways, positive regulation of phosphorylation, and others. Alterations within critical processes, such as phosphate metabolism, the regulation of phosphorylation, and immune system dynamics, have the potential to exert deleterious effects on the functionality of vital organs. Notably, organs integral to cardiometabolic health, including the liver, pancreas, heart, and kidney, are particularly susceptible to perturbations in these processes. This underscores the intricate relationship between the identified protein network and the modulation of key physiological pathways, thereby influencing the health of organs central to cardiometabolic function.

The major limitation of this study is the cross-sectional design, lacking longitudinal data or follow-up information on the patients, and the sample size. The latter limited our ability to perform gender-specific analysis, which could yield interesting results since we, and others, have previously observed a gender-specific association between sAAa and metabolic disorders.

Despite these limitations, our study is the first to examine the differences in circulating proteins between low and high sAAa and the link between these proteins and risk of cardiometabolic diseases. This study also highlights the significance of circulating proteome profiling in understanding the potential mechanisms underlying the relationship between sAAa and metabolic disorders such as obesity, insulin resistance, and diabetes. Furthermore, only high-risk overweight or obese individuals were included. Further validation and replication of our findings are warranted in larger and independent cohorts.

Obesity is known to influence metabolic and inflammatory pathways, which could modulate sAAa levels and their implications for cardiovascular health and inflammation. By concentrating on this specific group, we aim to provide more relevant insights into how sAAa levels are linked with cardiovascular and inflammatory markers in individuals who face distinct health challenges associated with excess body weight. We acknowledge that restricting the study to overweight and obese individuals may limit the generalizability of our findings to other populations, such as those with normal weight and helthy statu. Future research may include diverse populations to more understand the implications of sAAa levels across different weight categories and health statuses as well.

In conclusion, our study reveals discernible differential expression of circulating proteins among overweight/obese yet normoglycemic individuals stratified by low and high sAAa levels. The identified proteins exhibit direct or indirect involvement in numerous canonical pathways and cellular or molecular functions relevant to cardiometabolic disorders, including obesity, diabetes, hypertension, insulin resistance, stroke, and NAFLD. Delving deeper into the investigation of these proteins and associated pathways and functions holds the potential to elucidate the mechanistic foundations of the observed relationship between sAAa and metabolic disorders documented across diverse populations encompassing both adults and children.

Participants stratification into high and low sAAa subgroups, have elucidated potential associations between sAAa and markers of CVD and inflammation in overweight and obese individuals. These findings suggest that variations in sAAa levels may be linked with differences in cardiovascular and inflammatory markers among overweight and obese individuals. From a practical standpoint, our results could guide the development of targeted interventions for managing cardiovascular risk and inflammation in overweight and obese individuals. For instance, monitoring sAAa levels might offer a non-invasive approach to assess and manage these health conditions more effectively. Additionally, incorporating sAAa measurements into routine clinical practice could enhance the precision of risk assessments and the customization of treatment plans. Overall, our study highlights the potential of sAAa as a relevant biomarker in obesity-related health management and underscores the need for continued research to fully integrate these findings into clinical strategies.

## Data Availability

The raw data supporting the conclusions of this article will be made available by the authors, without undue reservation.
