# Supplementary material for: Differential expression of cardiometabolic and inflammation markers and signaling pathways between overweight/obese Qatari adults with high and low plasma salivary α-amylase activity
Source: Front Endocrinol (Lausanne). 2024 Oct 1;15:1421358. doi: 10.3389/fendo.2024.1421358 (PMC11473332; doi:10.3389/fendo.2024.1421358)
Supplement: Supplementary file 1 [file Table1.docx]

**Differential expression of cardiometabolic and inflammation markers and signalling pathways between overweight/obese Qatari adults with high and low plasma salivary α-amylase activity**

**Olfa Khalifa ^1^, Neyla Al-Akl ^1^** **, Abdelilah Arredouani ^2,3^**

^1^ Diabetes Research Center, Qatar Biomedical Research Institute, Hamad Bin Khalifa University, Qatar Foundation, PO Box: 34110, Doha, Qatar.

^2^ Diabetes Research Center, Qatar Biomedical Research Institute, Hamad Bin Khalifa University, Qatar Foundation, PO Box: 34110, Doha, Qatar.

^3^ College of Health and Life Sciences, Hamad Bin Khalifa University, Qatar Foundation, Doha, Qatar.

*Corresponding author: [aarredouani@hbku.edu.qa](mailto:aarredouani@hbku.edu.qa)

Qatar Biomedical Research Institute
Hamad Bin Khalifa University
PO Box: 34110
Tel: +974 445 42947
Fax: +974 445 41770
 Doha, Qatar

Number of words: 4534

Abstract: 287

Figures: 6

Tables: 2

Supplementary data table : 1

Supplemetray table 1 : Enrichment analysis and proteins with a VIP>1 identification to determine the enriched canonical pathways, diseases, and cellular and molecular functions

| **Traget proteins** | **ID** | **V1** | **130 prot** |
| --- | --- | --- | --- |
| **IL-1RT1 Cardiovascular III(v.6113)** | IL-1RT1 | 2.62421614 | 1 |
| **TLT-2 Cardiovascular III(v.6113)** | TLT-2 | 2.57051119 | 2 |
| **IL-1ra Cardiovascular II(v.5006)** | IL-1ra | 2.44175207 | 3 |
| **NCAM1 Cardiometabolic(v.3603)** | NCAM1 | 2.42660074 | 4 |
| **RARRES2 Cardiovascular III(v.6113)** | RARRES2 | 2.25919488 | 5 |
| **DPP4 Cardiometabolic(v.3603)** | DPP4 | 2.24747555 | 6 |
| **MMP-9 Cardiovascular III(v.6113)** | MMP-9 | 2.2077797 | 7 |
| **PGLYRP1 Cardiovascular III(v.6113)** | PGLYRP1 | 2.17564307 | 8 |
| **IL6 Cardiovascular II(v.5006)** | IL6 | 2.15573196 | 9 |
| **BTC Organ Damage(v.3311)** | BTC | 2.14384268 | 10 |
| **U-PAR Cardiovascular III(v.6113)** | U-PAR | 2.10646317 | 11 |
| **MB Cardiovascular III(v.6113)** | MB | 2.08666585 | 12 |
| **PTPRJ Organ Damage(v.3311)** | PTPRJ | 2.08558663 | 13 |
| **LEP Cardiovascular II(v.5006)** | LEP | 2.05586435 | 14 |
| **IGFBP6  Cardiometabolic(v.3603)** | IGFBP6 | 1.98631331 | 15 |
| **YES1 Organ Damage(v.3311)** | YES1 | 1.97234198 | 16 |
| **GP6 Cardiovascular III(v.6113)** | GP6 | 1.97140759 | 17 |
| **DECR1 Cardiovascular II(v.5006)** | DECR1 | 1.92470582 | 18 |
| **PD-L2 Cardiovascular II(v.5006)** | PD-L2 | 1.8925266 | 19 |
| **TNFRSF14 Cardiovascular III(v.6113)** | TNFRSF14 | 1.86104489 | 20 |
| **LCN2 Cardiometabolic(v.3603)** | LCN2 | 1.84792281 | 21 |
| **FABP4 Cardiovascular III(v.6113)** | FABP4 | 1.83665661 | 22 |
| **ENTPD2 Organ Damage(v.3311)** | ENTPD2 | 1.82735395 | 23 |
| **COMP Cardiometabolic(v.3603)** | COMP | 1.82616319 | 24 |
| **DCN Cardiovascular II(v.5006)** | DCN | 1.82471332 | 25 |
| **IL-1RT2 Cardiovascular III(v.6113)** | IL-1RT2 | 1.79765471 | 26 |
| **RETN Cardiovascular III(v.6113)** | RETN | 1.79261745 | 27 |
| **DPP7 Metabolism(v.3404)** | DPP7 | 1.78946028 | 28 |
| **HB-EGF Cardiovascular II(v.5006)** | HB-EGF | 1.78911019 | 29 |
| **NPTXR Metabolism(v.3404)** | NPTXR | 1.78494215 | 30 |
| **PCSK9 Cardiovascular III(v.6113)** | PCSK9 | 1.77273187 | 31 |
| **CA14 Organ Damage(v.3311)** | CA14 | 1.76458269 | 32 |
| **SNAP23 Metabolism(v.3404)** | SNAP23 | 1.76342189 | 33 |
| **THBS2 Cardiovascular II(v.5006)** | THBS2 | 1.74734481 | 34 |
| **CLEC1A Organ Damage(v.3311)** | CLEC1A | 1.73984691 | 35 |
| **SCF Cardiovascular II(v.5006)** | SCF | 1.7369822 | 36 |
| **LOX-1 Cardiovascular II(v.5006)** | LOX-1 | 1.7362912 | 37 |
| **KIT Cardiometabolic(v.3603)** | KIT | 1.73556017 | 38 |
| **PTX3 Cardiovascular II(v.5006)** | PTX3 | 1.73283818 | 39 |
| **CD40-L Cardiovascular II(v.5006)** | CD40-L | 1.71629494 | 40 |
| **PRTN3 Cardiovascular III(v.6113)** | PRTN3 | 1.71486331 | 41 |
| **SERPINA5 Cardiometabolic(v.3603)** | SERPINA5 | 1.71357134 | 42 |
| **CD84 Cardiovascular II(v.5006)** | CD84 | 1.70720993 | 43 |
| **PGF Organ Damage(v.3311)** | PGF | 1.68629523 | 44 |
| **ADM Cardiovascular II(v.5006)** | ADM | 1.67430383 | 45 |
| **REG4 Metabolism(v.3404)** | REG4 | 1.67193305 | 46 |
| **APLP1 Metabolism(v.3404)** | APLP1 | 1.66539481 | 47 |
| **CALCA Organ Damage(v.3311)** | CALCA | 1.66517138 | 48 |
| **SORT1 Cardiovascular II(v.5006)** | SORT1 | 1.65297832 | 49 |
| **MMP-3 Cardiovascular III(v.6113)** | MMP-3 | 1.64202624 | 50 |
| **TGFBI Cardiometabolic(v.3603)** | TGFBI | 1.63000404 | 51 |
| **AP-N Cardiovascular III(v.6113)** | AP-N | 1.62240431 | 52 |
| **CA13 Metabolism(v.3404)** | CA13 | 1.62038276 | 53 |
| **CNTN1 Cardiovascular III(v.6113)** | CNTN1 | 1.62006952 | 54 |
| **CD93 Cardiovascular III(v.6113)** | CD93 | 1.61523491 | 55 |
| **SRC Cardiovascular II(v.5006)** | SRC | 1.60537942 | 56 |
| **KLK6 Cardiovascular III(v.6113)** | KLK6 | 1.60307376 | 57 |
| **ROR1 Metabolism(v.3404)** | ROR1 | 1.58701673 | 58 |
| **CRKL Metabolism(v.3404)** | CRKL | 1.58484258 | 59 |
| **IL-17D Cardiovascular II(v.5006)** | IL-17D | 1.58186747 | 60 |
| **TF Cardiovascular II(v.5006)** | TF | 1.56959843 | 61 |
| **PRELP Cardiovascular II(v.5006)** | PRELP | 1.56829344 | 62 |
| **CD164 Metabolism(v.3404)** | CD164 | 1.56720314 | 63 |
| **EGFR Cardiovascular III(v.6113)** | EGFR | 1.55416689 | 64 |
| **ADGRE2 Metabolism(v.3404)** | ADGRE2 | 1.54340803 | 65 |
| **MPO Cardiovascular III(v.6113)** | MPO | 1.54266547 | 66 |
| **KIR3DL1 Organ Damage(v.3311)** | KIR3DL1 | 1.53819792 | 67 |
| **BID Organ Damage(v.3311)** | BID | 1.52735278 | 68 |
| **MERTK Cardiovascular II(v.5006)** | MERTK | 1.5253356 | 69 |
| **AXL Cardiovascular III(v.6113)** | AXL | 1.51880636 | 70 |
| **SOST Metabolism(v.3404)** | SOST | 1.51228996 | 71 |
| **BOC Cardiovascular II(v.5006)** | BOC | 1.50271754 | 72 |
| **CEACAM8 Cardiovascular II(v.5006)** | CEACAM8 | 1.49358953 | 73 |
| **STX8 Organ Damage(v.3311)** | STX8 | 1.49259373 | 74 |
| **LRIG1 Metabolism(v.3404)** | LRIG1 | 1.48946949 | 75 |
| **ACE2 Cardiovascular II(v.5006)** | ACE2 | 1.47029988 | 76 |
| **CDH5 Cardiovascular III(v.6113)** | CDH5 | 1.44092556 | 77 |
| **CDH1 Cardiometabolic(v.3603)** | CDH1 | 1.43507184 | 78 |
| **SLAMF7 Cardiovascular II(v.5006)** | SLAMF7 | 1.41692386 | 79 |
| **NPPC Organ Damage(v.3311)** | NPPC | 1.41075622 | 80 |
| **JAM-A Cardiovascular III(v.6113)** | JAM-A | 1.40692679 | 81 |
| **RNASE3 Metabolism(v.3404)** | RNASE3 | 1.40001199 | 82 |
| **AZU1 Cardiovascular III(v.6113)** | AZU1 | 1.38965551 | 83 |
| **TIE2 Cardiovascular II(v.5006)** | TIE2 | 1.38681098 | 84 |
| **CTRC Cardiovascular II(v.5006)** | CTRC | 1.38432225 | 85 |
| **DEFA1 Cardiometabolic(v.3603)** | DEFA1 | 1.38406134 | 86 |
| **PAPPA Cardiovascular II(v.5006)** | PAPPA | 1.35507738 | 87 |
| **ENG Cardiometabolic(v.3603)** | ENG | 1.34782247 | 88 |
| **STK4 Cardiovascular II(v.5006)** | STK4 | 1.34431645 | 89 |
| **SPON2 Cardiovascular II(v.5006)** | SPON2 | 1.34405794 | 90 |
| **CPA1 Cardiovascular III(v.6113)** | CPA1 | 1.33594446 | 91 |
| **TR Cardiovascular III(v.6113)** | TR | 1.32238778 | 92 |
| **BANK1 Organ Damage(v.3311)** | BANK1 | 1.29592568 | 93 |
| **IGFBP-7 Cardiovascular III(v.6113)** | IGFBP-7 | 1.27375097 | 94 |
| **RAGE Cardiovascular II(v.5006)** | RAGE | 1.26516107 | 95 |
| **TNF-R2 Cardiovascular III(v.6113)** | TNF-R2 | 1.25478054 | 96 |
| **TYMP Metabolism(v.3404)** | TYMP | 1.25407721 | 97 |
| **GAL Metabolism(v.3404)** | GAL | 1.24523843 | 98 |
| **CPB1 Cardiovascular III(v.6113)** | CPB1 | 1.23999098 | 99 |
| **PGF Cardiovascular II(v.5006)** | PGF | 1.23996735 | 100 |
| **CASP-3 Cardiovascular III(v.6113)** | CASP-3 | 1.22167 | 101 |
| **PARP-1 Cardiovascular II(v.5006)** | PARP-1 | 1.22030385 | 102 |
| **ANXA4 Metabolism(v.3404)** | ANXA4 | 1.21885448 | 103 |
| **TNF-R1 Cardiovascular III(v.6113)** | TNF-R1 | 1.21410441 | 104 |
| **FGF-21 Cardiovascular II(v.5006)** | FGF-21 | 1.20726563 | 105 |
| **ENPP7 Metabolism(v.3404)** | ENPP7 | 1.20407898 | 106 |
| **CNTN2 Organ Damage(v.3311)** | CNTN2 | 1.19307694 | 107 |
| **FBP1 Metabolism(v.3404)** | FBP1 | 1.19080697 | 108 |
| **FGR Organ Damage(v.3311)** | FGR | 1.18905332 | 109 |
| **SCGB3A2 Cardiovascular III(v.6113)** | SCGB3A2 | 1.18563586 | 110 |
| **TNFSF13B Cardiovascular III(v.6113)** | TNFSF13B | 1.17505474 | 111 |
| **LILRA5 Metabolism(v.3404)** | LILRA5 | 1.17200164 | 112 |
| **CA12 Organ Damage(v.3311)** | CA12 | 1.16799438 | 113 |
| **Ep-CAM Cardiovascular III(v.6113)** | Ep-CAM | 1.16166894 | 114 |
| **TYRO3 Metabolism(v.3404)** | TYRO3 | 1.14017744 | 115 |
| **DSG4 Organ Damage(v.3311)** | DSG4 | 1.11254545 | 116 |
| **TNFRSF10C Cardiovascular III(v.6113)** | TNFRSF10C | 1.11004951 | 117 |
| **ADGRG2 Metabolism(v.3404)** | ADGRG2 | 1.10155039 | 118 |
| **IL7R Cardiometabolic(v.3603)** | IL7R | 1.10091629 | 119 |
| **GHRL Metabolism(v.3404)** | GHRL | 1.08628656 | 120 |
| **KLK10 Metabolism(v.3404)** | KLK10 | 1.06204238 | 121 |
| **CTSD Cardiovascular III(v.6113)** | CTSD | 1.04735335 | 122 |
| **ICAM-2 Cardiovascular III(v.6113)** | ICAM-2 | 1.03129008 | 123 |
| **REN Cardiovascular II(v.5006)** | REN | 1.02607476 | 124 |
| **CSTB Cardiovascular III(v.6113)** | CSTB | 1.02520281 | 125 |
| **TIGAR Organ Damage(v.3311)** | TIGAR | 1.02234867 | 126 |
| **PTK7 Organ Damage(v.3311)** | PTK7 | 1.01697038 | 127 |
| **PDGF subunit A Cardiovascular III(v.6113)** | PDGF | 1.01128131 | 128 |
| **HO-1 Cardiovascular II(v.5006)** | HO-1 | 1.00369577 | 129 |
| **PRSS2 Cardiometabolic(v.3603)** | PRSS2 | 1.0013975 | 130 |
